# New Appliances and Things Medical

**Published:** 1909-06-26

**Authors:** 


### NEW APPLIANCES AND THINGS MEDICAL.

[We shall be glad to receive at our Office, 28 & 29 Southampton Street, Strand, London, W.C., from the manufacturers, specimens of all new preparations and appliances.]

### ENAMEL PAINTS FOR USE IN HOSPITALS.

Probably not the least difficult problem which architects, governing bodies, and superintendents of hospitals have to solve is the decoration of the walls of wards, staircases, waiting-halls, etc. On the one hand it is important that the decoration shall have, as far as is practicable, a certain soothing effect upon those to whom it is the only outlook for many days and nights together, and on the other hand cleanliness and asepsis must be fully provided for. Modern taste in decoration has led to the development of a number of so-called enamel paints, which, when dry, have something of the appearance of enamel. Samples of two kinds of paints of this type have been submitted to us—"Paripan," made by Messrs. Randall Bros., of Palmerston House, E.C., and "Robbialac," made by Messrs. Jenson and Nicholson, of Stratford, E. Both firms appear to have worked at the problem in very much the same manner. Both have realised that the ordinary plaster, however good it may be, and however well laid on, is naturally a harbourer of germs, in the very fullest sense of the term. It is very porous, and, in addition, is very rough. As Messrs. Jenson and Co. point out, an examination of any plaster wall under the microscope would show a number of hills and dales, and the dales form good refuge for germs. With both forms of paint, the plaster or woodwork—they are applicable to pretty well every kind of surface—is first covered with a paint specially prepared for the purpose, called by the makers of "Paripan" "filling," and by the makers of "Robbialac" "stopping." The object of both paints is the same as that of the under-paints used by the ordinary decorator, but are intended to do the work very much more thoroughly. They are to fill up the pores and to reduce all the inequalities to one smooth surface. The makers of "Robbialac" recommend that the surface be rubbed over, after "stopping" has been applied. With both forms of paint, after the "stopping" or "filling" has been thoroughly well worked in, and a smooth, hard, non-porous surface has been produced, a second coat, again specially provided for the purpose, and called by the makers of "Paripan" "Undercoat," and by the makers of "Robbialac" "Mat Finish," is applied. It is claimed that the coating of the second paint gives a better gloss than ordinary paint employed in high-class decorative work. The makers of "Robbialac" recommend that the surface shall be again rubbed over to make it smooth. Above the surface of the second coat a third is applied, called by the makers of "Paripan" "Glossy," and by Messrs. Jenson and Co. "Robbialac." The result of the proper application of the three coats, following on the careful "stopping" of the pores of the plaster, woodwork, etc., and of the careful preparation of the surface, is claimed to be a smooth, glossy surface that can be scrubbed or cleaned in any way that is convenient, that does not harbour dust or germs, and that is, in fact, in every way aseptic. "Paripan" has been used very largely at the London Hospital, and "Robbialac" has been used at the City of London Lying-in Hospital. Both are made in many colours, and Messrs. Jenson, in particular, keep a staff of skilled men for the purpose of arranging any desired colour.

### SUMNER'S MILK COVERS.

We have received for examination a sample cover, designed for the protection of vessels containing milk or other food-stuffs from contamination by flies. This is a square piece of gauze-like cloth, weighted at each corner, and easily placed over the mouth of vessels containing fluid or solid food. An accompanying pamphlet, which includes extracts from the M.D. Thesis on the "Causation and Prevention of Epidemic Infantile Diarrhœa," by Dr. V. J. Glover, explains the purpose and method of application of these simple and effective protectors. The covers are manufactured by R. Sumner and Co., Ltd., wholesale druggists, of Liverpool, in three sizes, 5 in. by 4½ in. at 4s. per 100 (small); 8 in. by 8 in. at 6s. per 100 (medium); and 10 in. by 10 in. at 8s. 8d. per 100 (large). Thus it is possible to purchase these covers to fit any ordinary size of food vessel. In addition to the covers for vessels, cot covers, 4 ft. by 3 ft., at 4s. 6d. per dozen, are supplied for the purpose of enveloping a baby's cot with fly-proof material and preventing the contamination by houseflies of the mouths of breast-fed infants. Milk-contamination by house-flies has by now been definitely established as one at least of the modes of dissemination of epidemic infantile diarrhœa; and these covers, simple in use and quite inexpensive, seem to be a reasonable means of checking the spread of this and other fly-borne diseases. We understand that the makers have produced these articles with the approval of Dr. Glover, and that copies of their explanatory pamphlet are now being distributed amongst the medical officers of health throughout the country, with a view to securing their approval and practical assistance. The covers seem to us to be quite efficient and to afford a cheap and accessible means of limiting the ravages of diseases spread by flies.

Professor M. J. M. Hill, Sc.D., F.R.S., late Fellow of St. Peter's College, Cambridge, has been elected Vice-Chancellor of the University of London for the year 1909-10. The best thanks of the Senate have been accorded to Sir William Collins, M.P., M.D., F.R.C.S., the retiring Vice-Chancellor, for the services which he has rendered to the University during his two years' tenure of that office.

